# Epistructured catechins, EGCG and EC facilitate apoptosis induction through targeting de novo lipogenesis pathway in HepG2 cells

**DOI:** 10.1186/s12935-018-0539-6

**Published:** 2018-03-21

**Authors:** Phuriwat Khiewkamrop, Pattamaphron Phunsomboon, Lysiane Richert, Dumrongsak Pekthong, Piyarat Srisawang

**Affiliations:** 1 Department of Physiology, Faculty of Medical Science, Naresuan University, Phitsanulok, 65000 Thailand; 2 Clinical Research Unit Floor 5 His Majesty’s 7th Cycle Birthday Anniversary 2, Faculty of Medicine, Naresuan University, Phitsanulok, 65000 Thailand; 3 KaLy-Cell, 20A rue du Général Leclerc, 67115 Plobsheim, France; 4 Laboratoire de Toxicologie Cellulaire, Université de Bourgogne Franche-Comté, EA 4267, Besançon, France; 5 Department of Pharmacy Practice, Faculty of Pharmaceutical Sciences, Naresuan University, Phitsanulok, 65000 Thailand

**Keywords:** Epistructured catechins, Epigallocatechin gallate (EGCG), Epicatechin (EC), Apoptosis, De novo lipogenesis (DNL), Carnitine palmitoyl transferase-1 (CPT-1)

## Abstract

**Background:**

Abnormally high expression of the mammalian de novo lipogenesis (DNL) pathway in various cancer cells promotes cell over-proliferation and resistance to apoptosis. Inhibition of key enzymes in the DNL pathway, namely, ATP citrate lyase, acetyl-CoA carboxylase, and fatty acid synthase (FASN) can increase apoptosis without cytotoxicity to non-cancerous cells, leading to the search for and presentation of novel selective and powerful targets for cancer therapy. Previous studies reported that epistructured catechins, epigallocatechin gallate (EGCG) and epicatechin (EC) exhibit different mechanisms regarding a strong inducer of apoptosis in various cancer cell lines. Thus, the current study investigated the growth inhibitory effect of EGCG and EC, on the enzyme expression and activity of the DNL pathway, which leads to the prominent activity of carnitine palmitoyl transferase-1 (CPT-1) mediating apoptosis in HepG2 cells.

**Methods:**

The cytotoxicity on HepG2 cells of EGCG and EC was determined by MTT assay. Cell death caused by apoptosis, the dissipation of mitochondrial membrane potential (MMP), and cell cycle arrest were then detected by flow cytometry. We further investigated the decrease of fatty acid levels associated with DNL retardation, followed by evaluation of DNL protein expression. Then, the negative inhibitory effect of depleted fatty acid synthesis on malonyl-CoA synthesis followed by regulating of CPT-1 activity was investigated. Thereafter, we inspected the enhanced reactive oxygen species (ROS) generation, which is recognized as one of the causes of apoptosis in HepG2 cells.

**Results:**

We found that EGCG and EC decreased cancer cell viability by increasing apoptosis as well as causing cell cycle arrest in HepG2 cells. Apoptosis was associated with MMP dissipation. Herein, EGCG and EC inhibited the expression of FASN enzymes contributing to decreasing fatty acid levels. Notably, this decrease consequently showed a suppressing effect on the CPT-1 activity. We suggest that epistructured catechin-induced apoptosis targets CPT-1 activity suppression mediated through diminishing the DNL pathway in HepG2 cells. In addition, increased ROS production was found after treatment with EGCG and EC, indicating oxidative stress mechanism-induced apoptosis. The strong apoptotic effect of EGCG and EC was specifically absent in primary human hepatocytes.

**Conclusion:**

Our supportive evidence confirms potential alternative cancer treatments by EGCG and EC that selectively target the DNL pathway.

## Background

Hepatocellular carcinoma (HCC), a primary malignancy of hepatocytes, is one of the fifth most common cancers and the third most common fatal cancer worldwide [1]. During the early stages of disease, liver resection is the most appropriate treatment for HCC patients. The other two common treatments for HCC include orthotopic liver transplantation (OLT) and chemotherapies, which still achieve low success rates with high resistance occurrence, depending on the stage of the disease. In addition, most of the chemotherapeutic agents for HCC patients, e.g., doxorubicin and gemcitabine have reported a high risk of serious side effects on normal non-cancerous tissue [2, 3]. Therefore, targeted treatments overcoming undesirable side effects with successful clinical outcomes are under consideration as alternative liver cancer therapies.

Nowadays, metabolic reprogramming is recognized as one of the special features of cancer cells. This reprogramming promotes sustained cell over-proliferation with suppression of cell apoptosis. In general, normal healthy cells express a low rate of glycolysis and generate energy primarily from oxidative phosphorylation (OXPHOS) in mitochondria. A reprogrammed metabolic pathway switches cancer cells to rely on a high rate of glycolysis, leading to elevation of pyruvate levels in the cytosol. This modified distinctive source of energy from normal cells is known as the “Warburg effect” [4, 5]. Besides enhanced glycolysis, OXPHOS is concomitantly under-operated in most cancer cells [6]. In addition, during the complexities of cancer development, the sustaining energy requirement under deprivation of nutrient supply stimulates an up-regulation of the de novo lipogenesis (DNL) pathway without depending on the extracellular fatty acid load [7]. The DNL pathway generates energy for cancer cells through β-oxidation and simultaneously provides precursors for cell membrane biosynthesis. ATP citrate lyase (ACLY), acetyl-CoA carboxylase (ACC), and fatty acid synthase (FASN) are key enzymes that regulate the conversion of a starting material citrate into newly synthesized fatty acids [8].

FASN produces saturated long chain fatty acids (LCFAs), primarily palmitic acid, from cytoplasmic substrates, including acetyl-CoA condensed with malonyl CoA in the presence of reductive NADPH activity. LCFAs are then converted into fatty acyl-CoA via acyl-CoA synthase (ACS) and translocated into the mitochondria crossing both the outer and inner membranes [7]. Carnitine palmitoyl transferase 1 (CPT-1) residing in the outer mitochondrial membrane esterifies fatty acyl CoA to acylcarnitine for subsequent translocation into the mitochondrial matrix by carnitine acylcarnitine translocase (CAT) for the ongoing β-oxidation pathway [9, 10]. Newly synthesized fatty acids from the DNL pathway play essential roles in cancer cell proliferation, metastasis, and resistance to cytotoxic states. Inhibition of the DNL pathway plays a regulatory role in apoptosis induction in various cancer cells. Targeting the DNL pathway by enzymatic inhibition can be considered an alternative therapeutic to cancers. Inhibition of ACLY can decrease cell proliferation in lung cancer cell lines [11]. Blocking the expression of ACC also enhances apoptosis in human glioblastoma cells [12]. Likewise, suppression of FASN expression and activity by cerulenin, C75, orlistat, or triclosan demonstrates apoptosis induction in many cancer cell lines [13–15]. Additionally, apoptosis promoted by inhibition of the DNL pathway has been suggested to be generated through a consequent suppression of CPT-1 activity. Increased accumulation of cytosolic fatty acyl-CoA levels is considered a mediator of DNL depletion that controls CPT-1 activity in apoptosis induction of cancer cells [16]. Not only synthetic inhibitors, but also many natural plant compounds have been described as having the ability to stimulate apoptosis by targeting the expression and activity of enzymes in the DNL pathway with an unclear understanding of the detailed underlying mechanisms [17, 18]. Interestingly, unlike cancer cells, inhibition of the DNL pathway lacks apoptosis effects on normal cells, suggesting a selective cytotoxic effect of targeting the DNL pathway in cancer cells [19].

Epigallocatechin gallate (EGCG) and epicatechin (EC) are both polyphenol compounds of the catechin group found in green tea (Camellia sinensis) with EGCG being more abundant than EC. Both compounds commonly possess high antioxidant activities [20]. Previous studies reported that EGCG exhibits alleviation effects in a variety of diseases, such as Alzheimer’s disease [21], Parkinson’s disease [22] and obesity [23]. EGCG has been found to be a strong inducer of apoptosis in various cancer cell lines, e.g., HCT116, HeLa, A549, and HepG2 cells [24–26]. Different mechanisms of EGCG regarding apoptosis induction in cancer cells have been reported. EGCG increases the expression of tumor suppresser genes, e.g., p53 [27], and suppresses receptors or signaling proteins involved in the proliferation pathways in various cancer cells, including the epidermal growth factor receptor (EGFR), human epidermal growth factor receptor-2 (HER2), insulin like growth factor receptor (IGF), and mitogen-activated protein kinase (MAPK) [28, 29]. Up-regulated expression of tumor suppressor genes, such as PTEN by EGCG leads to promoting apoptosis in human pancreatic cancer cells [30]. Moreover, a recent study suggests that EGCG suppresses the activity of phosphofructokinase, causing increased Bad expression and decreased Bcl-2 expression, which leads to increased apoptosis in hepatocellular carcinoma cells [26]. Indeed, the cytotoxic effects of EGCG’s targeting inhibition of FASN activity has been reported in breast cancer cells. However, the underlying mechanisms need to be explored in greater detail [31].

Unlike EGCG, EC has been less studied due to the smaller amount of EC presented in green tea. However, the effect of EC on apoptosis induction has been reported in the colon cancer cell line through increased expression of p53, leading to reduced Bcl-2 protein expression and increased Bax protein expression [32]. In addition, EC potentiates the effect of curcumin on the promotion of apoptosis in lung cancer cells [33]. Altogether, the underlying mechanisms of how EC activates cancer cell death are not clearly demonstrated. Targeting the DNL pathway by EC may clarify a potential anticancer effect of green tea leaves generated partly not only by the highest EGCG, but also by the lowest EC constituents.

The present study aimed to evaluate the apoptotic effects of EGCG and EC resulting from the inhibition of the DNL pathway in HepG2 cells. Depletion of fatty acid levels caused the suppression of CPT-1 activity, which was competitively inhibited by the increased malonyl CoA level. A low level of fatty acid production from the DNL pathway is known to show negative inhibition on ACC activity, producing a high malonyl CoA level. We suggest that inhibition of CPT-1 activities by EGCG and EC serves as the potential cause of apoptosis in HepG2 cells.

## Methods

### Cell culture

Hepatocellular carcinoma cells lines (HepG2) were obtained from American Type Culture Collection, (ATCC, Manassas, VA, USA). The culture of HepG2 cells was performed in Eagle’s minimum essential medium (EMEM) (Corning, USA) containing 10% fetal bovine serum (FBS) and 1% penicillin–streptomycin (100 units/ml of penicillin and 10 mg/ml of streptomycin) and incubated in a 5% CO_2_ incubator at 37 °C with humidity. Primary human hepatocytes were kindly provided by Prof. Dr. Lysiane Richert, KaLy-Cell, 20A rue du Général Leclerc, 67115 Plobsheim, France. Cells were cultured in human hepatocyte maintenance medium (Primacyt, Schwerin, Germany) containing 0.1 M dexamethasone (DEX), 50 mg/l gentamycin, 5% fetal calf serum and 4 mg/l of insulin. Cells were incubated in a 5% CO_2_ incubator maintained at 37 °C with humidity.

### Detection of cytotoxic effect by MTT assay

The anti-proliferative effects of EGCG and EC were investigated using a 3,-(4,5-dimethylthiazol-2-yl)-2,5-diphenyl tetrazolium bromide (MTT) assay (AMRESCO, Solon, OH, USA). In brief, cells were plated in a 96-well plate (SPL Life Sciences, Korea) at a density of 1 × 10^4^ cells/well. After treatment, MTT solution (1 mg/ml in PBS) was added and incubated in a CO_2_ incubator at 37 °C for 4 h. The mitochondrial reductase enzyme of healthy cells caused purple formazan crystal formation. Then, DMSO was added to dissolve this crystal. Absorbance was detected with a microplate reader (BioTek Instruments, Winooski, VT, USA) (BioTek) at 595 nm wavelength. Percentages of cell viability were calculated and compared with the control by Graph pad prism version 5.

### Apoptosis assessment by annexin V/PI staining

Stages of cell death through apoptosis were determined by double staining with annexin-V, and PI. Annexin-V staining was used to assess an apoptotic early stage of phosphatidylserine (PS) translocation to the extracellular cell membrane compartment while both annexin-V and PI staining detected an apoptotic late stage [34]. HepG2 cells were cultured in a 24-well plate at a density of 1 × 10^6^ cells/well overnight. Then, both adherent and floating cells were harvested and stained with Alexar Flour 488 annexin-V and PI by Alexar Flour 488 annexin-V/Dead Cell Apoptosis Kit (Life Technologies, Invitrogen, Grand Island, NY, USA). Apoptotic rates were analyzed by FACScalibur flow cytometry using CellQuestPro software (Becton–Dickinson, Franklin Lakes, New Jersey, USA).

### A mitochondrial damage dependent apoptosis assessment by JC-1 staining

The level of ∆Ψm in HepG2 cells was examined by FACScalibur flow cytometry using 5,6-dichloro-2-[3-(5,6-dichloro-1,3-diethyl-1,3-dihydro-2*H*-benzimidazol-2-ylidene)-1-propenyl]-1,3-diethyl-, iodide (JC-1), and a cationic mitochondrial membrane potential fluorescence probe (Invitrogen, USA). A high polarization state of ∆Ψm makes the positive charges of JC-1 accumulated in the electronegative interior of the mitochondrial matrix and exhibits red fluorescence emission at 590 nm while the disruption of ∆Ψm shows decreasing red dye accumulation in the mitochondrial matrix and increasing green monomeric form from the cytoplasm and emits green fluorescence at 530 nm. The red/green fluorescence intensity ratio represents a dissipation of ∆Ψm [18]. HepG2 cells were cultured in a 24-well plate at a density of 1 × 10^5^ cells/well and allowed to attach overnight. CCCP was used as a positive control to verify the depolarization status of ∆Ψm. After treatment, both adherent and floating cells were incubated with JC-1 in a CO_2_ incubator and detected by FACScalibur flow cytometry using excitation at 488 nm and emission 585/42 filter. Data were analyzed using CellQuestPro software.

### Investigation the cell cycle by PI staining

Induction of cell cycle arrest during apoptosis was examined by flow cytometry using propidium iodine (PI) binding with DNA. HepG2 cells were seeded at a density of 1 × 10^6^ cells/35-mm culture dish and incubated overnight for adherence. Following the treatment, cells were harvests and fixed by 70% ethanol at 4 °C overnight. RNase A (AMRESCO, Solon, OH, USA) was then added. Finally, cells were stained with PI and cellular DNA contents were measured by FACScalibur flow cytometry, and data were analyzed using CellQuestPro software.

### Bcl2 activity assay

Induction of mitochondrial disruption in apoptosis is controlled by expression and activity of the anti-apoptotic Bcl2 protein, which is assessed by MUSE Bcl2 Activation Dual Detection Kit (Millipore, USA) [35]. HepG2 cells were cultured at a density of approximately 1 × 10^6^ cells in a 35-mm culture dish and incubated 24 h for adherence. During the following treatment, cells were collected, a fixative was added, and then the cells were incubated on ice. Cell pellets were subsequently re-suspended, and an antibody cocktail was added containing anti-phospho-Bcl2 (Ser70), Alexar Flour 555, anti-Bcl-2, and PECy5. Flow cytometry was performed by the bench top MUSE Cell Analyzer (Millipore, USA), and the percentage of the Bcl-2 activity compared with controls (100%) was calculated by Graph pad prism version 5.

### Determining the intracellular ROS generation by DCFH-DA assay

Induction of oxidative stress by reactive oxygen species (ROS) product was detected by FACScalibur flow cytometry using a 5-(and-6)-chloromethyl-29, 79-dichlorodihydrofluorescein diacetate fluorescence dye (CM-H2DCFDA) (Molecular Probes, USA). HepG2 cells were cultured in a 24-well plate at a density of 1 × 10^5^ cells/well for 24 h. In the following treatment, cells were harvested and re-suspended in 10 µM of CM-H2DCFDA. Then, stained cells were detected by FACScalibur flow cytometry using excitation and emission filter at 485 and 525 nm, respectively. Data were analyzed using CellQuestPro software.

### Western blotting

HepG2 cells were seeded in a 35-mm culture dish with a density of approximately 1 × 10^6^ cells/dish and allowed 24 h to attach. Cells were harvested and lyzed by M-PER Mammalian Protein Extraction Reagent (Thermo Fisher Scientific, USA) containing the proteinase inhibitor cocktail (Thermo Fisher Scientific, USA). Protein from cell lysate was collected and concentration quantified by BCA Assay Reagent (Thermo Fisher Scientific, USA). Equal amounts of proteins per lane were separated by 8–12% (SDS) polyacrylamide gel electrophoresis and transferred to PVDF membranes. Then, the membranes were incubated with RAPIDBLOCK solution (Thermo Fisher Scientific, USA). Membranes were then incubated with anti-FASN (Abcam, USA), anti-ACC (Merck Millipore, USA), and anti-ACLY (Cell Signaling Technology, USA) and then exposed to horseradish peroxidase-conjugated goat anti-rabbit secondary antibody (Life Technologies, Invitrogen). β-actin was used as an internal control (Cell Signaling Technology, USA). Finally, protein bands were visualized by LuminataTM Forte Western HRP Substrate (Merck Millipore, USA) and detected by CCD camera (Chemiluminescence Image Quant LAS 4000; GE Healthcare Life Sciences, Pittsburgh, PA, USA). Percentages of relative expression levels of protein/actin were calculated by Image J software version 1.46.

### Quantification of free fatty acid product from DNL activity

Long chain free fatty acid products of the DNL pathway were evaluated using the Free Fatty Acid Quantification Kit (US, Biological, MA, USA). HepG2 cells were stored at a density of 1 × 10^6^ cells/35 mm in a culture dish and incubated overnight. After treatment, cells were collected with chloroform-Triton-X 100 (1% Triton X 100 in pure chloroform), centrifuged, and finally air dried to remove chloroform from the samples. The dried lipid was dissolved by fatty acid assay buffer, and Acyl-CoA synthase was then added to each sample. The fluorescence probe and the enhancer were added to the reaction. Fluorescence signal was detected at Ex/Em 535/590 nm. Percentages of long chain free fatty acid level were calculated and compared with the control by Graph pad prism version 5.

### CPT-1 activity assay

Spectrophotometry was used to determine CPT-1 activity as had been reported previously [17]. In brief, cells were seeded at a density of 2 × 10^6^ cells in a 60 mm culture dish and incubated overnight. Then, they were exposed to green tea polyphenols, and the cells were harvested. After being lyzed, collected mitochondrial protein extraction was added with 0.01 mM palmitoyl CoA and l-carnitine. CPT-1 activity was measured by microplate reader at 412 nm wavelength. Percentages of mitochondrial CPT-1 activity were calculated and compared with the control by Graph pad prism version 5.

### Statistical analysis

The data of the three independent experiments are shown as mean ± SEM and paired one-way analysis of variance (ANOVA) or Student’s t test with Turkey’s post hoc analysis was used to determine all experiments. The mean of the control was compared to consider statistically significant differences at p < 0.05. All data used Graph Prism Software version 5 for analysis.

## Results

### EGCG and EC decreased cell viability in HepG2 cell lines

**Fig. 1:**
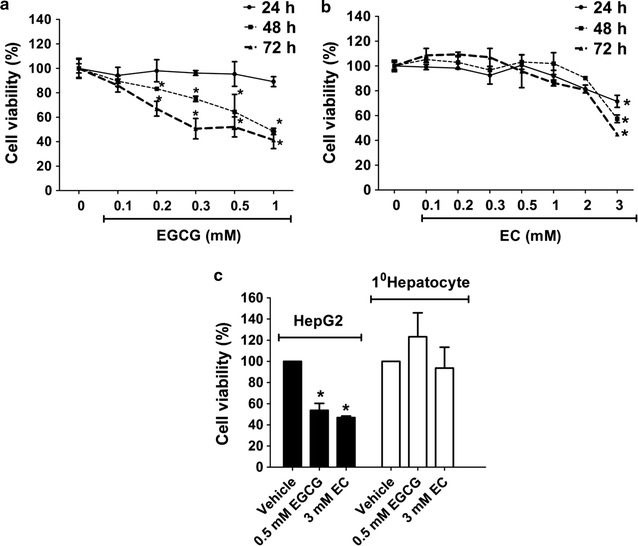
The cytotoxic effects of EGCG and EC on HepG2 cells and primary hepatocytes. Cells were treated with EGCG and EC at indicated dose and times. Cells incubated with 0.1% DMSO without EGCG or EC were identified as the control. Cell viability was assessed by MTT assay. **a**, **b** Cell viability of HepG2 cells and **c** HepG2 cells and primary hepatocytes were treated with EGCG or EC at IC50 concentration for 72 h. Data from at least three independent triplicated experiments are presented as mean ± SD, n = 9, *p < 0.05, denoting significant differences compared with the control

Anti-cancer characteristics are presented in green tea polyphenols. We first measured the cell viability by MTT assay in HepG2 cells. After treatment with EGCG and EC, we found decreasing cell viability with dose and time, as shown in Fig. 1a, b. The lowest IC50 of EGCG and EC exposure were 0.5 and 3.0 mM at 72 h exposure, respectively. This suggests more potent anticancer activity of EGCG than EC in HepG2 cells. To determine selective cytotoxic effects of EGCG and EC, primary normal human hepatocytes were used. At IC50 doses of EGCG and EC for 72 h incubation period, no cytotoxic effect was found in primary hepatocyte cells as shown in Fig. 1c.

### EGCG and EC induced cell death with mitochondrial-mediated apoptosis in HepG2 cells

**Fig. 2:**
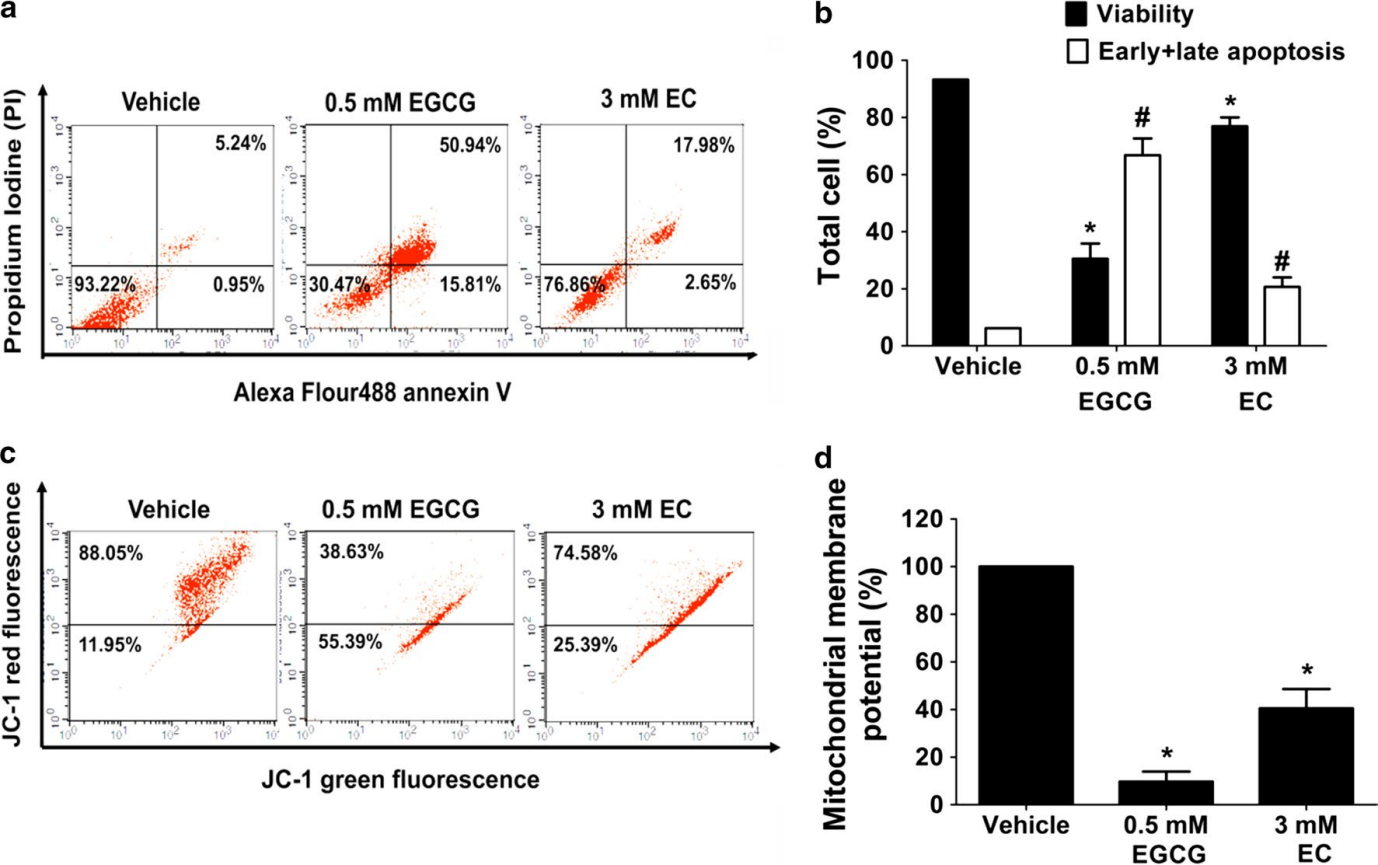
The apoptotic induction mediated by disruption of ∆Ψm with effects of EGCG and EC in HepG2 cells. Cells were treated with EGCG and EC for 72 h. Controls were incubated with 0.1% DMSO. **a** The population of apoptotic cells was determined by flow cytometry and depicted as representative flow cytometry scatterplots. **b** The histogram shows percentages of the distribution of viable and total apoptotic (early and late) cells measured by double staining of annexin-V and PI. **c** The ∆Ψm was investigated by JC-1 dye staining and detected by flow cytometry. **d** The histogram shows percentages of red and green fluorescent intensity ratios representing ∆Ψm compared with 100% of the control vehicle. Data from at least three independent triplicated experiments are presented as mean ± SD, n = 3, *p < 0.05 and ^#^p < 0.05, denoting significant differences compared with the viable and apoptotic control groups, respectively

Cytotoxic effects of EGCG and EC inducing apoptosis pathway were investigated by a flow cytometer. HepG2 cells were treated for 72 h at indicated IC50 concentrations obtained from the MTT assay. One of the hallmark characteristics of apoptotic cell death in the early stage was assessed by positive staining with annexin V to phosphatidylserine (PS) translocated to the extracellular membrane and negative staining with PI while the late stage was positively stained with both annexin-V and PI as shown in Fig. 2a, b. EGCG and EC statistically significant increased the total apoptosis rate approximately from 6 to 67% and 21% (at p value 0.05), respectively, suggesting cytotoxic apoptotic effects of EGCG and EC on HepG2 cells.

Further evaluation of the apoptosis feature was by mitochondrial disruption. As Fig. 2c shows, mitochondrial aggregated JC-1 dye represents a high ∆Ψm, while cytoplasmic monomeric form represents a decreased ∆Ψm. Figure 2d shows the histogram of percentage of ∆Ψm expressed as 100% in the control. After treatment with EGCG and EC, we found a statistically significant loss of ∆Ψm in HepG2 cells (at p value of 0.05). Thus, these results provide additional indication that mitochondrial disruption accounts for apoptosis cell death induced by EGCG and EC in HepG2 cells.

### EGCG and EC induced cell cycle arrest in HepG2 cells

**Fig. 3:**
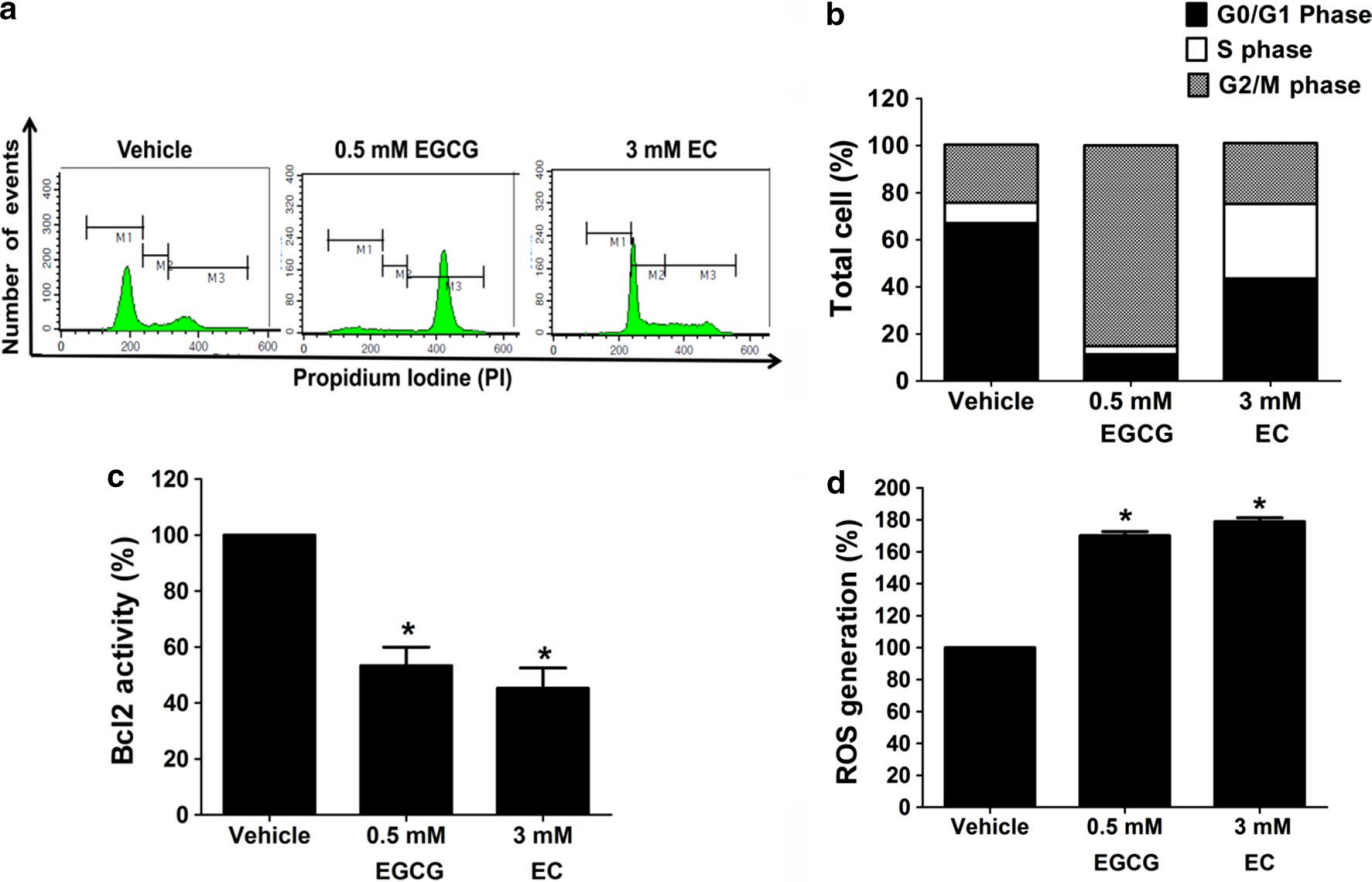
EGCG and EC induced cell cycle arrest, Bcl2 activity, and ROS production in HepG2 cells. Cells were treated with EGCG and EC for 72 h. Controls were treated with 0.1% DMSO. **a** Cell cycle arrest was investigated by PI staining and detected by flow cytometry. Representatives of the cell population during cell cycle phases and **b** quantitative percentages of all cell cycle phases are expressed in the histogram. **c** Activity of Bcl2 was determined by MUSE Cell Analyzer flow cytometry and percentage calculated for Bcl2 activity compared with 100% of the vehicle control. **d** ROS production was determined by CM-H_2_DCFDA fluorescent dye, detected by flow cytometry, and percentages calculated for ROS product compared with 100% of control. Data are expressed as mean ± SD from at least a triplicate of n = 3, *p < 0.05, denoting significant differences compared with the control

Cell cycle arrest correlated with apoptosis progression after treatment with EGCG and EC was evaluated. Figure 3a, b show that HepG2 cells treated with EGCG and EC have a significantly elevated population of cells in G2/M and S phases arrested compared with the control.

### EGCGC and EC decreased Bcl2 activity in HepG2 cells

Apoptosis is known to result from inhibition of Bcl2 and increase of Bax protein activities that facilitate mitochondrial damage [36]. Figure 3c shows that HepG2 treated with EGCG and EC exhibited statistically significant decreased activity of Bcl2 from 100 to 53.32% and 45.23%, respectively, confirming EGCG and EC target Bcl2 activity in HepG2 cells.

### EGCG and EC increased ROS production in HepG2 cells

At the same time, enhanced ROS generation is suggested to stimulate apoptosis in cancer cells [37]. HepG2 cells treated with EGCG and EC resulted in statistically elevation of intracellular ROS production form 100% of control to 170.13 and 178.93%, respectively, as shown in Fig. 3d. These results suggest a correlation of ROS generation and apoptosis in HepG2 cells.

### EGCG and EC suppressed DNL protein expression and fatty acid production in HepG2 cells

**Fig. 4:**
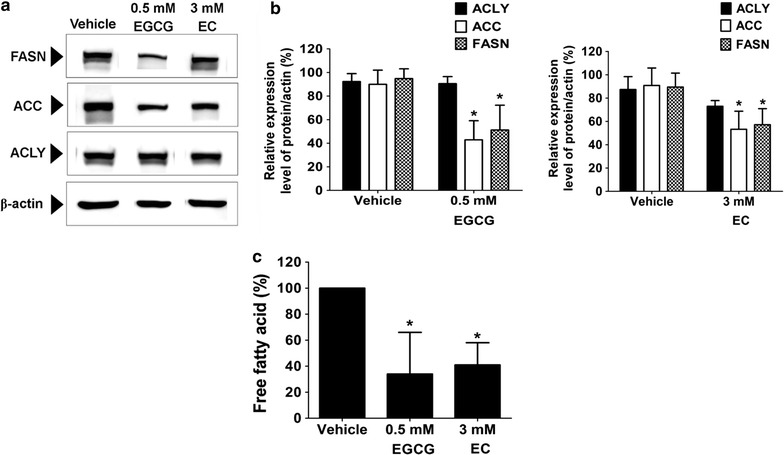
EGCG and EC decreased the DNL pathway in HepG2 cells. Cells were treated with EGCG and EC for 72 h. Control cells were treated with 0.1% DMSO. **a** The expression of enzymes in the DNL pathway was determined by immunoblotting and **b** percentages calculated for protein expression are compared with 100% of control. **c** The histogram shows effects of EGCG and EC decreased the percentage of free fatty acid levels compared with the control. Data are expressed as mean ± SD from at least a triplicate of n = 3, *p < 0.05, denoting significant differences compared with the control

In the present study, we investigated effects of EGCG and EC on expression of proteins in the DNL pathway, including FASN, ACLY, and ACC. The immunoblotting assay in Fig. 4a shows the expression of the proteins FASN, ACC, and ACLY. FASN and ACC were decreased following EGCG and EC treatment but not for ACLY. Similarly, no change of ACLY expression level was detected while the expression levels of ACC and FASN proteins were significantly decreased approximately to 42 and 51% following EGCG and to 53 and 57% following EC treatment, respectively, as shown in Fig. 4b. However, this decrease of protein expression was consistent with the reduction of the free fatty acid level, as shown in Fig. 4c. These results provide additional evidence that the targeted DNL pathway is considered to be the mechanism underlying apoptosis induction by EGCG and EC in HepG2 cells.

### Accumulation of malonyl-CoA as a consequence of suppression of the DNL pathway by EGCG and EC inhibited CPT-1 activity which leads to activate apoptosis in HepG2 cells

**Fig. 5:**
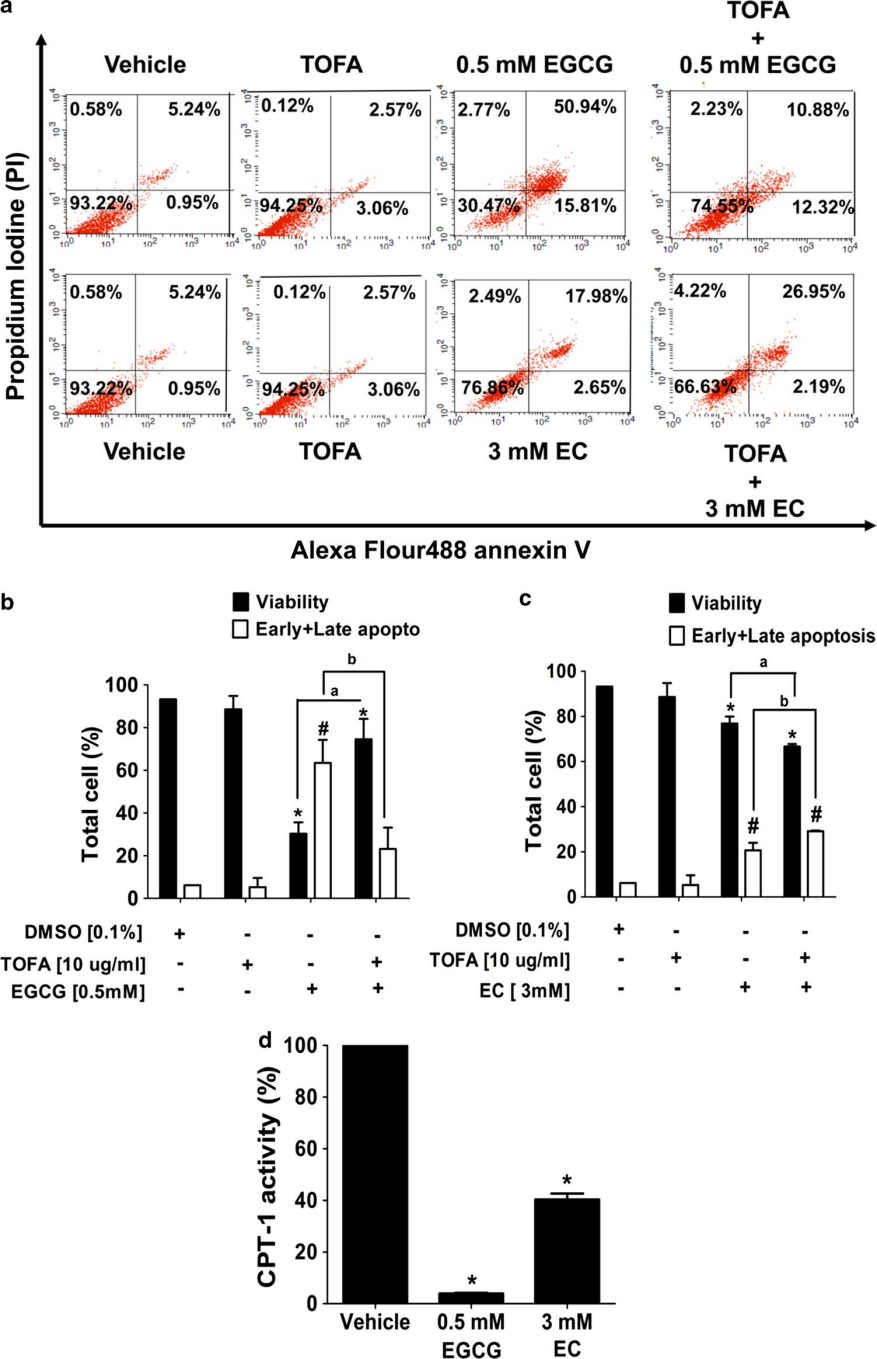
Apoptotic induction by EGCG and EC correlates with malonyl-CoA accumulation that consequently results in CPT-1 activity suppressing in HepG2 cells. Cells were treated with EGCG and EC for 72 h. TOFA at 10 µg/ml was preincubated and then cells were treated with EGCG and EC for 72 h. Control cells were treated with 0.1% DMSO. **a** The population of apoptotic cells was investigated by double staining of Annexin V/PI, determined by flow cytometry, and depicted as representative flow cytometry scatterplots. **b**, **c** The histogram shows percentages of cell viability and total apoptosis. **d** The percentages of the CPT-1 activity were measured after EGCG and EC treatment for 72 h. Data are expressed as mean ± SD from at least a triplicate of n = 3, *p and ^#^p < 0.05, denoting significant differences compared with the viable and apoptotic control groups, respectively

In a previous study, it has been reported that the inhibition of FASN and the reduction of free fatty acid production reduces the inhibition on ACC activity, leading to induced malonyl CoA accumulation. This accumulation is considered to be one of the major causes of apoptosis induction in cancer cells [18]. Figure 5a–c show that the competitive inhibitor of ACC, 5-(tetradecyloxy)-2-furoic acid (TOFA) restored cell viability and ameliorated apoptosis from EGCG treatment, which suggests that the accumulation of malonyl CoA from EGCG-suppressed fatty acid appears to be the predominant cause of apoptosis. In contrast, the inhibition of ACC did not rescue apoptotic cells from EC treatment, demonstrating that suppression of the DNL pathway by EC results in apoptosis through an independence of malonyl CoA accumulation. Additional results shown in Fig. 5d support our postulation that the suppression of the DNL pathway by EGCG causes HepG2 apoptosis, and that suppression inhibits the CPT-1 activity via an accumulation of malonyl CoA. Contrary to this, EC inhibited the DNL pathway directly, causing suppression of the CPT-1 activity and leading to apoptosis in HepG2 cells.

## Discussion

The roles of EGCG and EC, which are two important active polyphenol compounds in green tea leaves, have been most commonly studied focusing on anti-cancer effects. Administration of EGCG and EC inhibits cell proliferation and induces apoptosis in various cancer cells [26, 32]. This study demonstrated that EGCG and EC selectively decreased cell viability and increased cell death through the apoptosis pathway in HepG2 cells by targeting the DNL pathway. Decreased expression of enzymes in the DNL pathway, ACC and FASN, contributes to the reduction of fatty acid synthesis and promotes apoptosis in HepG2 cells. Moreover, apoptotic activity resulted from inhibition of Bcl2 activity, consequently generating damaged mitochondrial membrane potential. In addition, we suggest that the reduced DNL pathway causing suppression of CPT-1 activity may contribute to decreased Bcl2 activity, which in turn causes mitochondrial dependent apoptosis in HepG2 cells. Indeed, dissipated mitochondrial membrane potential can increase ROS production, which also implicates the induction of apoptosis in HepG2 cells.

In contrast to normal cells, one of the hallmark features of various cancers cells is the continuous requirement of upregulated biological synthesis of cell membranes and cellular proteins to support a high rate of cell proliferation while the apoptosis rate is suppressed [38]. Up-regulation of enzyme activities in the glycolysis pathway provides more of the needed ATP than OXPHOS. This change is due to reduction in pyruvate entering the TCA cycle, resulting in decreased energy generation from the OXPHOS pathway [36]. In the meantime, up-regulation of the enzyme lactate dehydrogenase (LDH) can convert pyruvate to lactate, thereby leading to further decreased OXPHOS activity in cancer cells [26]. To accommodate the reduction of energy production from glucose metabolism, the DNL pathway is known to be upregulated to provide alternative energy and starting materials for cellular biosynthesis. In contrast, the expression of FASN, a key enzyme in the DNL pathway, in normal cells is underdetectable except in lipogenic tissues, including liver, breast, colon, and prostate where excessive carbohydrates are stored in lipid or triglyceride forms to be used in starvation situations [39]. However, cancer cells still have higher levels of FASN than those of lipogeneic tissues [17]. Overexpression of FASN in cancerous cells is regulated through multiple signaling pathways, including growth factor/growth factor receptors, such as the epidermal growth factor receptor (EGFR), human epidermal growth factor-2 (HER2), and hypoxia conditions [40]. FASN expression in cancer cells is also activated by activation of tumor suppressor gene downstream pathways, for example, phosphoinositide-3 kinase (PI3K)/v-akt murine thymoma viral oncogene homolog (AKT)/rapamycin complex 1 (mTORC1) and mitogen-activated protein kinase (MAPK) signaling pathways, leading to enhanced tumorigenesis [39, 41]. AKT-activated mTORC1 expression causes an activation of sterol regulatory element-binding protein 1 (SREBP1) that leads to stimulation of the lipogenic enzyme expression, including FASN and ACC [42]. Moreover, one of the main causes of the reprogramming pathway in tumors arises from hypoxia conditions. The hypoxic environment can promote stabilization of HIF1α which leads to increased expression of SREBP1 and FASN in the cancer cell line [40].

The present study found an inhibitory effect of EGCG on the expression of DNL enzymes. However, the underlying mechanisms were not further examined. EGCG has been previously reported to inhibit activation of IGF/IGFR activity in liver and colon cancer cells [28, 43]. Inhibitory effects of EGCG have focused on the expression of EGFR, HER-2, and androgen receptors, leading to decreased expression of PI3K/AKT/mTOR signaling in colon and prostate cancer cells [44, 45]. In addition, EGCG generates the AMPK expression that results in the inhibition of mTORC1/SREBP1 axis expression, leading to decreased FASN expression in human hepatoma cells [46]. Moreover, green tea extraction and EGCG can reduce HIF1α expression in Hela and HepG2 cell lines [47]. Inhibition of HIF1α expression by Oroxylin A can decrease SREBP1 and FASN expression in the colon cancer cell line [40]. Subsequently, reduction of free fatty acid through inhibited FASN expression by cerulenin and C75 can induce a cytotoxic effect in the breast cancer cell line.

An increase in the malonyl CoA level can suppress β-oxidation from the fatty acid pathway by inhibiting the activity of CPT-1 at the outer membrane of mitochondria [48]. Moreover, previous study has also demonstrated that the inhibiting activity and expression of CPT-1 by etomoxir, a CPT-1 inhibitor, can promote apoptosis in leukemia cells [49]. This evidence suggests that the suppression of FASN facilitates apoptosis by causing an inhibition of the CPT-1 activity. The present study found a consistent result regarding EGCG and EC influence on apoptosis induction in HepG2 cells, where suppressed expression of FASN leads to the inactivation of the CPT-1 activity. Reports on breast cancer cells also support our suggestion that the inhibitory effect of EGCG on the FASN activity contributes to an inhibition of the CPT-1 activity. This effect promotes apoptosis and induces inhibition of HER-2, AKT, and ERK1/2 signaling expression [31]. We further used an inhibitor of ACC to ascertain that suppression of the DNL pathway by EGCG produces an inhibition of the CPT-1 activity through malonyl CoA accumulation in HepG2 cells. The combination of FASN and malonyl CoA inhibition reduced apoptosis cell death instead of FASN inhibition alone. Our evidence was supported by a previous study that found that inhibition of ACC by TOFA (ACC inhibitor) can reduce fatty acid levels but does not promote cytotoxicity in cancer cell lines [50]. Thus, accumulation of malonyl CoA level may represent a major cause of apoptosis cell death rather than a reduction of free fatty acid level per se. Although the suppression of the DNL pathway by EC produces an inhibition on CPT-1 activity, the accumulation of malonyl CoA was not the dominant inhibitor of the CPT-1 activity, which implies direct inhibitory activity of suppressed fatty acid on CPT-1 activity.

So far, we have analyzed the effect of the suppressed CPT-1 activity on mitochondrial apoptosis induction. We found a correlation of Bcl2 deficiency and CPT-1 activity suppression by EGCG and EC treatment, so the CPT-1 suppression activity could inhibit Bcl2 activity. A previous study demonstrated that the inhibition of the CPT-1 activity through knocking down the expression of FASN in breast cancer cells results in promoting an increase of ceramide levels [51]. Inhibition of the CPT-1 activity by etomoxir confirms the result of suppressing the conversion of fatty acyl CoA to fatty acylcarnitine, an accumulated fatty acyl CoA, which influences increased ceramide levels [52]. Accumulated cytosolic fatty acyl CoA can condense with serine to generate 3-ketosphinganine, which is then metabolized by ceramide synthase to produce ceramide [53]. As a result, an increased ceramide level can activate apoptosis by enhancing the expression of the pro-apoptotic protein Bcl2 which interacts with protein 3 (BNIP3), as reported in breast cancer cells. However, abolishing the apoptosis by decreased ceramide level and an increased BNIP3 protein under inhibition of ceramide synthase confirms apoptosis induction by ceramide [51]. Thus, inhibition of Bcl2 protein expression comes from the inhibited CPT-1 activity that results in increased ceramide synthesis. An overexpressed BNIP3 is a mediator of ceramide and Bcl2 [54, 55]. Similar studies reported that the inhibition of Bcl2 expression activates a translocation of Bax from cytoplasm to mitochondria to bind with endophilin B1 and then triggers mitochondrial damage-mediated apoptosis [56, 57]. An important study reported that EGCG promotes apoptosis by decreasing the combination of Bcl2 to Bax protein, resulting in activation of the translocation of Bax to cause mitochondrial damage [26].

**Fig. 6:**
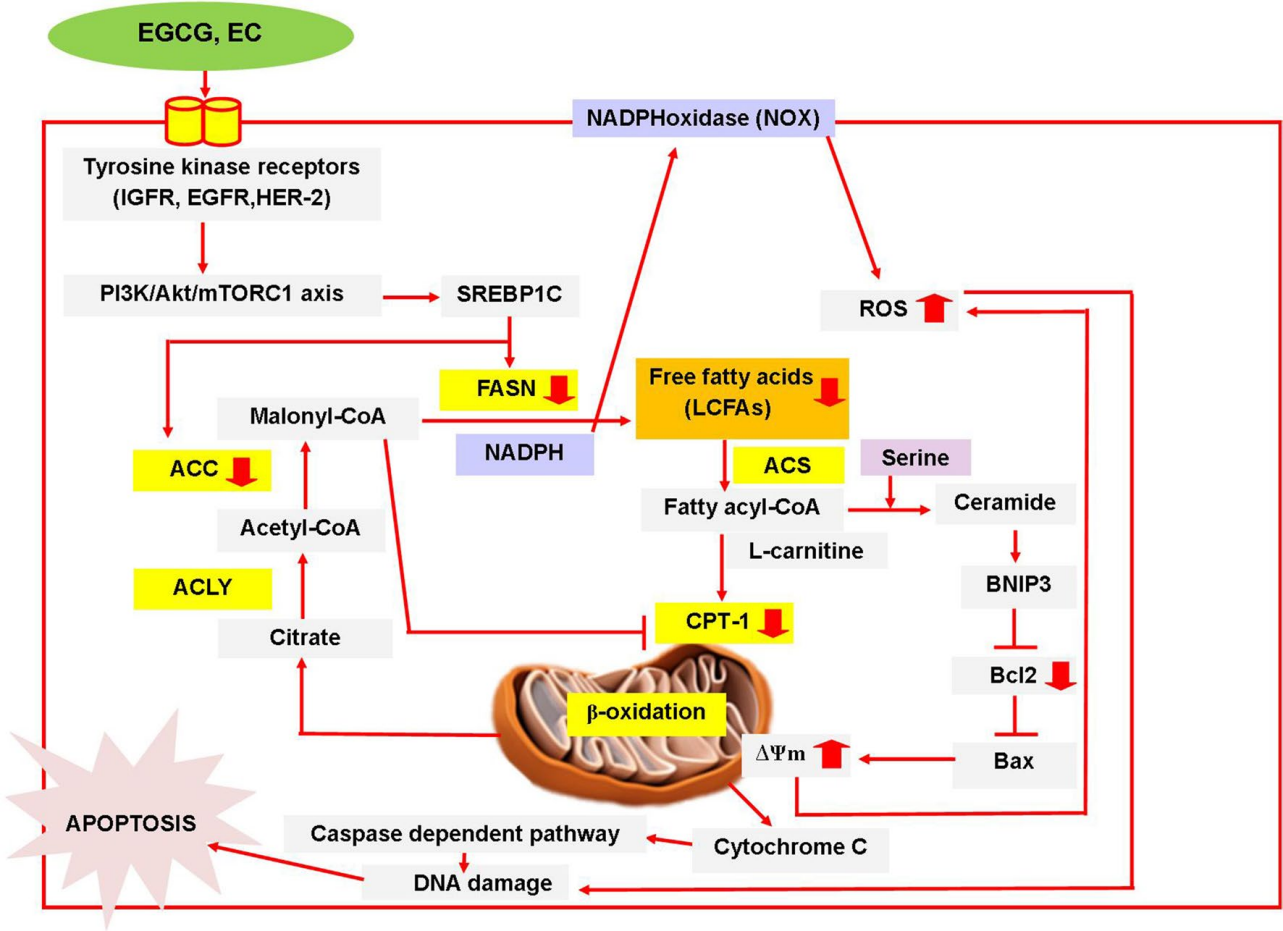
Schematic diagram illustrating the principal mechanism of EGCG and EC in the DNL pathway-mediated apoptosis in HepG2 cells. EGCG and EC decrease the expression of enzymes in DNL pathway including ACC and FASN, which is then results in reducing free fatty acid levels. The possible mechanisms of the anti-DNL pathway of EGCG and EC may be resulted from inhibition of EGCG on the tyrosine kinase receptor mediating PI3K/Akt/mTORC1/SREBP1 axis [28, 39, 42]. Subsequently, reduction of free fatty acid exhibits inhibition of mitochondrial CPT-1 activity, thereby contributing to disruption of lipid β-oxidation [18]. This inhibition results in promoting an increase of ceramide levels. As a result, ceramide can promote mitochondrial dependent apoptosis by activating the interaction of Bcl2 and BNIP3 [16, 54]. In addition, accumulation of ROS due to mitochondrial dysfunction and NADPH accumulation contributes to apoptosis in HepG2 [60]

Reactive oxygen species (ROS) is known to specify reactive chemical species containing oxygen that are classified into two groups: free radical and non-free radicals [58]. ROS in cancer cells is generated from mitochondrial respiration processes and plasma membrane NADPH oxidase complexes [59]. Mitochondrial dysfunction by EGCG has been identified as a source of ROS generation that promotes apoptosis in hepatoma cells [60]. In addition, inhibition of FASN activity and expression has been reported to exert apoptosis through enhanced intracellular ROS level [61, 62]. The present study showed that EGCG and EC promoted ROS production in HepG2 cells, suggesting ROS contributes to apoptosis. Several reports state that ROS promotes apoptosis in cancer cells. It has been reported that the apoptotic effect of EGCG is regulated by elevation of oxidative stress level in colon cancer cells. Inhibition of ROS by *N*-acetyl-l-cysteine (NAC), a precursor of glutathione, can abolish the effect of EGCG-induced apoptosis in the HT-29 cell line [63]. ROS can inhibit Ca^2+^-ATPase, leading to depletion of Ca^2+^ stored in the endoplasmic reticulum (ER). This depletion of calcium promotes ER stress and triggers apoptosis [64]. Moreover, increased ROS production also induces the release of cytochrome c from mitochondria into the cytoplasm, resulting in activation of an apoptosis cascade in prostate cancer cells [65]. The principal mechanism of EGCG and EC in the DNL pathway-mediated apoptosis in HepG2 cells is schematically diagrammed in Fig. 6.

## Conclusions

The current study demonstrates that suppression of the DNL pathway in HepG2 cancer cells by EGCG and EC decreases activity of CPT-1. An accumulation of ceramide as a consequence promotes apoptosis through decreasing Bcl2 protein activity. This research proposes anticancer agents of natural products that trigger apoptosis through targeting inhibition of key enzymes in the DNL pathway. A selective cytotoxic effect in cancer of epistructured catechins will offer a beneficial therapeutic approach to future studies of alternatives to anticancer treatments.

## Abbreviations

EGCG: epigallocatechin gallate
EC: epicatechin
CPT-1: carnitine palmitoyl transferase-1
DNL: de novo lipogenesis
HCC: hepatocellular carcinoma
OXPHOS: oxidative phosphorylation
LCFAs: long chain fatty acids
ACLY: ATP-citrate lyase
ACC: acetyl-CoA carboxylase
FASN: fatty acid synthase
MUFAs: monounsaturated fatty acids
ROS: reactive oxygen species
TOFA: 5-(tetradecyloxy)-2-furoic acid
